# Restructuring healthcare services, routines and procedures on reproductive medicine based on respect for differences

**DOI:** 10.5935/1518-0557.20230061

**Published:** 2024

**Authors:** Nelson Antunes Junior, Andrea Giannotti Galuppo, Jonathas Borges Soares, Sidney Glina

**Affiliations:** 1 Clinica Pluris, Director - São Paulo, SP, Brazil; 2 ALFA Project, Director - São Paulo, SP, Brazil; 3 Clinica Pluris, Researcher - São Paulo, SP, Brazil; 4 Discipline of Urology at Centro Universitário - FMABC - Santo André, SP, Brazil

**Keywords:** assisted reproduction, homosexuality, transgender

## Abstract

Although the term homosexuality was removed from the International Classification of Diseases and *trans* identities from mental disorders, these classifications promote the pathologizing of homosexuality. The direct consequence is discrimination, which adds to the difficulty in carrying out accurate information related to the LGBT population and makes it very difficult to organize public policies suited to their needs. An important issue is related to the limited access of that population to assisted reproduction techniques, when compared to traditional families. The desire for same sex couples and transgender persons to have biological children is reportedly the same as for cisgender persons, but parenthood can be a much greater endeavor both medically and psychologically for them. The right to health includes freedom to control one’s health and body, including sexual and reproductive issues. Despite these difficulties, we are living in a period of great social progress that increases access to assisted reproduction among novel patient populations. With legalization of gay marriage, individuals and couples who identify as lesbian, gay, bisexual and transgender, may seek to begin or expand their families with assisted reproduction technologies. Therefore, the aim of this review was to assist in the restructuring of healthcare services, routines and procedures, mainly related to reproductive medicine, in order to promote changes in values based on respect for differences. In conclusion, the healthcare personnel of fertility centers should undergo specific training and preparations to meet the specific demands of the LGBT patient population and to overcome communication barriers.

## INTRODUCTION

Every person has a sex, sexual orientation, and a gender identity, but the three are independent. Both sexual orientation and gender identity can be fluid and change over time (Obedin-Maliver & Makadon, 2016). A person’s desire for intimacy with people of the same gender, lesbians and gay men, or both men and women in the case of bisexual people, are terms that refer to sexual orientation (Obedin-Maliver & Makadon, 2016). Already gender identity is related to whether individuals identify themselves as a man, a woman, or one of many other genders (Obedin-Maliver & Makadon, 2016). The development of male or female characteristics occurs during infant life and childhood, together with gender self-awareness (Hembree *et al*., 2017). Interactions with parents, peers, and the environment are essential in the process of cognitive and affective learning (Hembree *et al*., 2017). Although the biological control of gender identity is not yet fully understood, the scientific factors behind genetic sex are fairly well understood (de Ziegler & de Sutter, 2021). Distinct from gender identity, the genetic sex, determines the development of inner and outer sex organs and the correspondent hormonal production (de Ziegler & de Sutter, 2021). In most cases, there is functional harmony between gender identity and genetic sex, but divergence may exist generally without, but sometimes with, disorders of sexual development (Amir *et al*., 2020; de Ziegler & de Sutter, 2021). A disorder related to sexual development is gender dysphoria, which is defined as the occurrence of a mismatch between genetic sex and gender identity (de Ziegler & de Sutter, 2021). It is present in approximately 0.5% of the population, which means 35 million individuals of today’s global world population, and it is important to remark that the true prevalence of gender dysphoria is probably underestimated (Meriggiola & Gava, 2015; de Ziegler & de Sutter, 2021).

Although gender dysphoria has been labeled as a psychological disorder, it has not been proved by neurophysiological nor psychological studies, which a satisfactory explanation (Meriggiola & Gava, 2015). The term homosexuality was removed from the International Classification of Diseases in 1990, and *trans* identities was taken off the mental disorders in 2019 (WHO, 2019). That kind of classification promoted the pathologizing of homosexuality, and the direct consequence of it was discrimination (United Nations. Human Rights Council, 2022). As a direct consequence of discrimination there is the difficulty in carrying out an accurate survey of the size of the LGBT population in each country and in the world, which makes it very difficult to organize public policies suited to their needs. Recent surveys show a wide variation in the number of people who identify themselves as lesbian/gay (Greenfeld & Seli, 2016). Apparently the homosexual population in countries like the United States, Canada, United Kingdom and Norway was estimated at 1% of the total population (Flores *et al*., 2016; Greenfeld & Seli, 2016). Even without knowing the size of the LGBT population, following WHO guidance, Brazil organized an LGBT policy based on the recognition of the effects of discrimination and exclusion in the health-disease process of the LGBT population (Brazil, 2013). Brazil’s LGBT policy aimed at changes in the social determination of healthcare to reducing inequalities (Brazil, 2013).

And precisely that search for the reduction of inequalities during the first decade of the twenty-first century, related to the progress in the field of LGBT rights, impose for several countries the review of legislation that regulates family formation, filiation and marriage (Imaz, 2017). Despite this social evolution concerning LGBT rights in many countries, in others homosexuality remains illegal and is severely repressed (Greenfeld & Seli, 2016). Also, according to the United Nations (UN), some countries continue to classify homosexuality as an illness and in almost all countries trans persons are treated as if they were sick or disordered (United Nations. Human Rights Council, 2022). And it is this type of conceptualization that renders reports from several authors about the discrimination or disparities in the healthcare setting related to LGBT individuals (Meriggiola & Gava, 2015; Greenfeld & Seli, 2016; Jin & Dasgupta, 2016). The disparities observed in general healthcare for LGBT people is also perceived in the limited access of that population to assisted reproduction techniques, when compared to traditional families (Kim, 2020).

The desire for same sex couples and transgender persons to have biological children is reportedly the same as for cisgender persons (Greenfeld & Seli, 2016; Amir *et al*., 2020). LGBT people are increasingly considering their fertility options as reproductive techniques, although the majority of births still occur among heterosexual married couples (Amir *et al*., 2020; Kim, 2020). However, parenthood can be a much greater endeavor both medically and psychologically for them, and the main barriers to treatment are prohibitive costs, homophobia and geographical location (Greenfeld & Seli, 2016; United Nations. Human Rights Council, 2022). In an effort to address the disparities experienced by LGBT individuals insofar as fertility is concerned, the Ethics Committee of the American Society for Reproductive Medicine and the American Congress of Obstetricians and Gynecologists have argued that providing competent care for LGBT persons at fertility centers is an ethical duty (ACOG, 2012; Ethics Committee of American Society for Reproductive Medicine, 2013; 2015). The right to healthcare includes freedom to control one’s health and body, including sexual and reproductive freedom, non-consensual medical treatment, as well as entitlements (WHO, 2019). But despite the difficulties it is important to notice that we are living in a period of great social progress that allows for growing access to assisted reproduction among novel patient populations (Kim, 2020). Therefore, the aim of this review was assist in the restructuring of healthcare services, routines and procedures, mainly related to reproductive medicine, in order to promote changes in values based on respect for differences.

## MATERIALS AND METHODS

This study was conducted as a review of articles published between 2012 and 2022. For this purpose, we searched for papers on Google Scholar, Scopus, PubMed, Science Direct, and ISI databases.

## RESULTS AND DISCUSSION

According to the United Nations. Human Rights Council (2022), the right to healthcare must be ensured to all without discrimination, but it is well established that sexual orientation and gender identity are prohibited grounds of discrimination. One example of that are the data presented by Light *et al*. (2014), showing that most transgender men used nonphysician providers and nonhospital birth locations more frequently than the general public, which could expose transgender men to higher risk of peripartum complications (Thornton & Mattatall, 2021). The visibility of health issues for the LGBT population, in Brazil, started in the 1980s, facing the HIV/AIDS epidemic, when the Ministry of Health, together with social movements joined forces to advocate for the rights of gay groups (Brazil, 2013). Although the AIDS epidemic caused the healthcare system to focus its priorities on transvestites and transsexuals, giving this group some visibility, it is currently known that these people’s health problems are much more complex and their demands are numerous (Brazil, 2013).

### Reproductive health

The recognition of the complexity of LGBT health demands a cross-sectional organization, that encompasses all areas of the Ministry of Health, such as the production of knowledge, social participation, promotion, attention and care (Brazil, 2013). Ultimately, the healthcare system must provide equality of opportunity for people to enjoy the highest attainable level of health (United Nations. Human Rights Council, 2022). But, despite the social differences related to the access to healthcare services between the overall patient and the LBGT patient, the disparities are also associated with biological needs (Pirtea *et al*., 2021). It is important that medicine incorporate gender diversity into routine care, there are several issues to be addressed by the physician considering LBGTs patients, for example, breast cancer screening after chest reconstruction surgery for transgender men, or how and when to do prostate examinations for transgender women, how to care for transgender men who desire to be or are pregnant, and the indiscriminate and unguided use of hormones (Brazil, 2013; Obedin-Maliver & Makadon, 2016).

### Gender-Affirming Hormone Therapy (GAHT)

Considering the transexual population the use of gender-affirming hormone therapy (GAHT) is common and not always performed under medical supervision; therefore, the consequences related to long-term GATH on hormone-sensitive organs, like ovaries and breasts, and the metabolism in general must be observed (Pirtea *et al*., 2021). When GAHT is done without medical supervision the occurrence of self-medication with high doses of hormones is an aggravating factor in the health of these people (Brazil, 2013). Regarding the gender-incongruent adolescents, the primary risks of pubertal suppression may promote adverse effects on bone mineralization, compromised fertility, and unknown effects on brain development (Hembree *et al*., 2017). It was already described in adults the association between the use of female hormones and the occurrence of stroke, phlebitis, myocardial infarction, among other disorders, resulting in deaths or important sequelae (de Ziegler & de Sutter, 2021). Usually, the breasts respond to GAHT therapy with a transformation of the glandular tissue for a fibrous tissue, and that change can be related to premalignant and/or malignant transformation (de Ziegler & de Sutter, 2021). The ovaries also respond to hormonal treatment by simulating certain characteristics encountered in polycystic ovary syndrome (de Ziegler & de Sutter, 2021).

The treatment of transgender persons should be individualized and multidisciplinary, and it consists of medical treatment aimed at suppression of assigned gender sexual characteristics, gender-affirming hormone treatment (GAHT) and/or sex reassignment surgery (SRS) (Baram *et al*., 2019). While these treatment options often help alleviate the symptoms of gender dysphoria, decrease depression and increase self-esteem, there are significant fertility risks that should be considered prior to commencing the transition process (Gorin-Lazard *et al*., 2013; Baram *et al*., 2019). It is clear that transgender persons should be encouraged to consider fertility preservation before starting GAHT and undergo a break in treatment of at least 3 months if the hormone therapy has already begun (Amir *et al*., 2020). Therefore, there will be a growing need for specialized care for transgender people specially because of the increased awareness of gender dysphoria and its relationship with transition procedures (de Ziegler & de Sutter, 2021). Persons considering hormone use for gender affirmation need adequate information about this treatment concerning the general health and about specific fertility effects and options for fertility preservation should be discussed (Hembree *et al*., 2017).

### Assisted reproduction for LGBTQIA+ people

The initial regulation of assisted reproductive techniques reflected the dominant ideas of parental suitability, and the access to infertility treatment was orientated in terms of heterosexual exposure to unprotected sex without pregnancy (Smietana *et al*., 2018). All the treatment protocols were developed considering only heterosexual couples and/or donors or surrogates standing in for them (Smietana *et al*., 2018). However, individuals and couples who identify themselves as lesbian, gay, bisexual and transgender (LGBT), with legalization of gay marriage, may seek to begin or expand their families with assisted reproduction technologies (James-Abra *et al*., 2015; Leung *et al*., 2019; Kim, 2020). The development of an equality legislation in the UK prohibited refusal of fertility treatment based on sexual orientation according to the Protected Characteristics (Bodri *et al*., 2018). And in Europe and the USA, the right to access fertility treatments for singles, lesbian and gay couples as well as transgender people was recognized by the American Society for Reproductive Medicine (Ethics Committee of American Society for Reproductive Medicine, 2013). In Brazil a resolution from the Federal Board of Medicine determined that the assisted reproduction techniques must be available for all capable persons who request it, since they are in full agreement and duly informed in accordance with the current legislation (CFM Resolution No. 2.320/2022).

However, the lack of formal medical education related to LGBT healthcare issues associated with the variety of assisted reproductive procedures available may afford significant barriers for healthcare providers to assist LGBT individuals or couples with quality care (Stern *et al*., 2002; Obedin-Maliver & Makadon, 2016). Accordingly, documented disparities between LGBT patients and the overall patient population persist. Specific examples in ART include lesbian or bisexual women receiving fertility services at half the rate of heterosexual white women (Blanchfield & Patterson, 2015; Jin & Dasgupta, 2016). LGBT patients repeatedly expressed a desire for more information regarding fertility options and access to reproductive health care providers who respect, support, and understand their gender identity (Light *et al*., 2014). A LGBT-friendly team, with a safe and welcoming environment at all service levels, including administrators, mental health workers and medical providers, increases compliance with fertility preservation and treatments (Amir *et al*., 2020). Fertility treatment centers increasingly recognize issues unique to gay men and women and are increasingly welcoming (Greenfeld & Seli, 2016).

**Figure 1:**
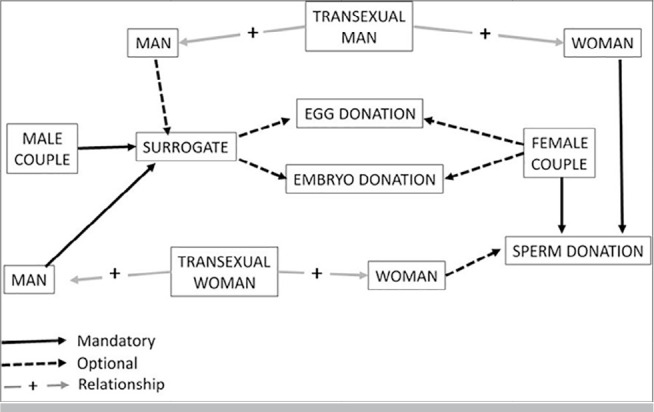
Simplified scheme showing the different possibilities of procedures according to the structure of the couple.

In order to familiarize patients with the reproductive procedures, many fertility centers have online education on their websites (Jin & Dasgupta, 2016). Fertility services are essential for LGBT couples and a simple tool as online information can facilitate decision making concerning reproductive options (Jin & Dasgupta, 2016). It is known that the availability of information in accessible language empowers patients to be able to make informed decisions about personal reproductive options (Jin & Dasgupta, 2016). The consultation must include an assessment of the couples’ understanding of the treatment (Figure 1), their familial support, and their ability to handle the stress of reproductive technology (Greenfeld & Seli, 2016).

### Same sex couples

Basically, the assisted reproduction techniques for lesbian or bisexual women in same-sex relationships include intrauterine insemination and in vitro fertilization (IVF) or intracytoplasmic sperm injection (ICSI) with donor sperm (Baram *et al*., 2019). In the cases of female same sex couples with indication of IVF or ICSI, the oocytes were collected from the woman identified by the specialist as the one with major chances of producing quality embryos, based on physiological parameters. And when the IVF or ICSI was the procedure of choice there is the possibility of a shared pregnancy, when the embryo obtained from the fertilization of a woman’s oocyte(s) is transferred to her partner’s uterus (CFM Resolution No. 2.320/2022). The shared pregnancy or intra-partner egg donation was already reported as an acceptable, successful and safe treatment option offering good obstetrical and perinatal outcomes (Bodri *et al*., 2018). However, it is important to remark that there are differences among countries according to marriage, registered partnership, legal parentship and adoption rules that can limit the reproductive autonomy of lesbian couples wishing to conceive (Bodri *et al*., 2018). Countries like Brazil, Belgium, Finland, Ireland, the Netherlands, Spain, Portugal and the UK allow this treatment option without any restriction, since same-sex marriage is allowed, lesbians are eligible for all forms of assisted reproduction techniques, and intra-partner egg donation is not restricted (Bodri *et al*., 2018; CFM, 2022).

For gay or bisexual men in same-sex relationships will always be necessary to have a traditional surrogate or oocyte donation, and a gestational surrogate (Baram *et al*., 2019). It is important to remark that in Brazil the surrogate mother needs to be a volunteer and must be blood related to the fourth degree of one of the partners (CFM Resolution No. 2.320/2022). In other countries, like Spain, USA and Canada surrogacy is a complicated and expensive process (Imaz, 2017). Unfortunately, the options available for men in same-sex relationships and to single men are certainly more expensive when compared with the possibilities available to single women and female same-sex couples (Imaz, 2017). Therefore, only a few gay couples will be able to access parenting via assisted reproduction techniques, not only in terms of money, but also of time, availability, and the different resources that the surrogacy option requires (Imaz, 2017).

### Pubertal children trans-individuals

In order to help transgender people to minimize gender dysphoria the use of hormone therapy is an option, since it enables the achievement of physical changes consistent with their perceived gender (Amir *et al*., 2020). That kind of therapy should not be used only for adults but also for pubertal children (Tanner stage ≥2) (Hembree *et al*., 2017). Specially for pubertal children the effect of the hormonal therapy pausing puberty provides a significant extra time, extremely necessary for the exploration of gender identity (Guss & Gordon, 2022). The hormone therapy for pubertal children is based on the hormonal gonadotropin-releasing hormone agonists (GnRHa) administration for puberty blockade to prevent the development of secondary sexual characteristics (Amir *et al*., 2020; Panagiotakopoulos *et al*., 2020). A GnRHa promote the downregulation of the pituitary-gonadal axis leading to the inhibition of sex steroid secretion, which ceases the development of pubertal changes (Guss & Gordon, 2022).

However, hormonal therapy is not free of risks, and it could promote higher risk of developing cancer and impairment of fertility potential in adulthood (Panagiotakopoulos *et al*., 2020). Thus, the pubertal blockade should be discussed, not only with the patient, but also with their caregivers, and all the information about the benefits and risks should be carefully considered (Panagiotakopoulos *et al*., 2020). Once the adolescent identify himself or herself as a transgender after the GnRHa treatment it is possible to initiate the gender-affirming hormone (GAH) therapy (Amir *et al*., 2020). The GAH therapy consists of a testosterone treatment for transgender males and estrogen for transgender females; and it can be initiated at 14-16 years of age (Amir *et al*., 2020; Guss & Gordon, 2022). And after 18 years of age, the gender-affirming surgery can be done, in case of transgender women the upper body surgery includes breast enlargement and in transgender men the removal of breast tissue. Also, a lower body surgery can be performed to remove reproductive organs and reconstruct them into the desired organs (Amir *et al*., 2020; Guss & Gordon, 2022).

Though the hormonal therapy, GAH and affirming surgery are helpful tools that enable transgender individuals live healthier lives, when those therapies are considered by pubertal children is important to regard fertility preservation options before treatment onset. Although the administration of GnRHa will temporarily impair spermatogenesis and oocyte maturation, in order to recover ovary and testicle function, it is necessary to delay or temporarily discontinue GnRHa therapy (Hembree *et al*., 2017). Once the hormonal therapy is interrupted, it causes the prompt resumption of the pituitary-gonadal axis (Guss & Gordon, 2022). But this option is often not preferred because it enables the development of later stages of puberty with significant development of secondary sex characteristics (Hembree *et al*., 2017). At present there is no available data concerning the time required for restoration of spermatogenesis or for spontaneous ovulation for later fertility (Hembree *et al*., 2017).

Some specialists propose a discussion about the lack of knowledge of pubertal children about the potential effects of hormonal interventions that can hamper the decisions about fertility preservation (Hembree *et al*., 2017; Smietana *et al*., 2018). Those arguments have been used to deny surgery or hormones to prepubertal trans youth (Smietana *et al*., 2018). That is why parents and other members of the adolescent’s support group must be informed about the future consequences and help the children make an assertive decision (Hembree *et al*., 2017). In case they choose to preserve fertility before gender affirming therapies it is important to look for a reproduction specialist. The technologies available today, considering that they are young people, are semen cryopreservation for transgender women and oocyte cryopreservation for transgender men. The procedure for semen cryopreservation is simpler and of low cost when compared to oocyte cryopreservation, because to collect the oocytes, a stimulation protocol is required, followed by a surgical retrieval.

### Adults trans-individuals

A transgender man is someone who sees himself as a man but has a female genetic sex (Obedin-Maliver & Makadon, 2016). The pattern therapy is based on a multidisciplinary approach consisting of testosterone replacement associated with sex reassignment surgery, which includes mastectomy and hysterectomy combined with bilateral oophorectomy (Wierckx *et al*., 2012). Clinical studies have demonstrated the efficacy of different hormonal therapies based on androgen preparations focusing on inducing masculinization in transgender males (Wierckx *et al*., 2012; Meriggiola & Gava, 2015). During the use of those hormonal therapies occur the reversible loss of fertility (Meriggiola & Gava, 2015; Amir *et al*., 2020). Although fertility can be restored after discontinuing the hormones, it was reported that it can take up to 24 months for the reproductive function to return to normal (Light *et al*., 2014). However, associated with the restoration of fertility the sexual secondary characteristics suppressed by the treatment resurge, which can be a problem for many individuals (Light *et al*., 2014). Even considering that effect from discontinuing the hormonal treatment there are results demonstrating that transgender men desire children and are willing and able to conceive, carry a pregnancy, and give birth (Light *et al*., 2014; Obedin-Maliver & Makadon, 2016). It is recommended that, prior to any testosterone treatment or surgery, transgender men who want to have genetically related children consider either embryo or oocyte cryopreservation (Obedin-Maliver & Makadon, 2016; Amir *et al.,* 2020). In cases when the transgender man has undergone previous surgical procedures like a hysterectomy or ovariectomy, there are three available options for the preservation of reproductive possibilities oocyte banking, embryo banking and banking of ovarian tissue at the time of surgery (Amir *et al*., 2020). Therefore, a recent study showed that the majority of transsexual men had not considered freezing their germ cells prior to begin sex hormone therapy, and despite 20% had considered it, they had never looked for a specialist to discuss this issue (Wierckx *et al*., 2012).

The majority of transsexual women were treated with hormone therapy with anti-androgens and estrogens, sometimes associated with gender affirming surgery (Wierckx *et al*., 2012). It is important to know that there seems to be an association between estrogen exposure, androgen deprivation, and altered testicular function (Adeleye *et al*., 2019). The feminizing hormone therapy will lead initially to hypo-spermatogenesis and eventually to azoospermia, which can be irreversible over time (Adeleye *et al*., 2019). That is why the use of gender affirming hormonal medication at the time of specimen collection is negatively associated with parameters of semen quality (Adeleye *et al*., 2019). Therefore, both hormonal and surgical interventions negatively affect the male reproductive system (Wierckx *et al*., 2012). However, patients who decided to cryopreserve sperm prior to gender affirming therapy had better semen parameters within the WHO reference range, compared to transgender women that started hormone treatment previously to the time of specimen collection (Adeleye *et al*., 2019).

Considering that fertility preservation is recommended by cryopreservation of sperm samples, before undergoing hormonal therapy for future use in assisted reproductive techniques (ART) (Wierckx *et al*., 2012; Adeleye *et al*., 2019). If transsexual women have a female partner, they can procure children through intrauterine insemination, in vitro fertilization or intracytoplasmic sperm injection, based upon the sperm quality after thawing (Wierckx *et al*., 2012). Reproductive options for transsexual women with a male partner are more difficult as they need oocyte donation as well as a surrogate mother (Wierckx *et al*., 2012). But in cases were the patient already initiated the hormonal therapy it was demonstrated that after months of discontinuation of the hormonal medication, it is possible for transgender women to produce a specimen with parameters good enough for intrauterine insemination or to potentially conceive spontaneously (Adeleye *et al*., 2019). However, when the discontinuation of gender affirming medication is not an option, but the individual is able to ejaculate, it may be possible to produce specimens for use via *in vitro* fertilization instead of intrauterine insemination (Adeleye *et al*., 2019). Although the ejaculation itself would not be a predictive factor of active spermatogenesis, since there is a large intra-patient variation in semen parameters between samples (Adeleye *et al*., 2019). Despite the recommendation of fertility preservation before starting hormonal therapy in order to keep sample quality for future use, several factors can be related to a reduction of motivation towards fertility preservation in transwomen (Wierckx *et al*., 2012). The main factor seems to be that many transsexual women favor a fast transition over future fertility concerns (Wierckx *et al*., 2012).

### Paradigm shift for the laboratory

Considering that the LGBT population approaching medical care has been growing, there is an increasing need for research aiming to specialize health care according to their specific needs (Rodriguez-Wallberg *et al*., 2021). In this sense the increasingly seeking to access reproductive services shed light on the optimal way to provide effective care to these patients (Leung *et al*., 2019). Therefore, the role of reproduction researchers and embryologists is linked to the development of protocols and biotechnologies that fit the profile of this new group of patients. LGBT couples in most cases are not infertile, so probably in the next few years some adapted protocols should be developed, in order to optimize the laboratory results and cost of the procedures (Leung *et al*., 2019). Thinking of a possibly similar group of patients, a better comparison group would be oncology fertility patients, since they are not infertile but still need the assistance of a reproduction center (Leung *et al*., 2019).

Within the LGBT patients there is a specific group, the transgender male or female, who need special attention, since their reproductive potential is suppressed by gender-affirming surgery, and it can also be impaired by hormonal treatments (Sermondade *et al*., 2021). Although very important for the choice of laboratory techniques and protocols, only a few studies have investigated the gamete quality of transgender women and/or men, and most of them have a small sample of patients. Considering the transgender women, semen analysis is the most important tool for assessing sperm quality and help detect infertility-related problems. We know that the transition hormonal therapy with estrogens can impair fertility causing sperm alterations, such as lower sperm quality, which could complicate and limit the efficiency of reproductive procedures (Sermondade *et al*., 2021; Rodriguez-Wallberg *et al*., 2021). Lower sperm quality in individuals that began gender affirming hormonal therapy presented high rates of sperm abnormalities, lower total sperm count and sperm concentration (Rodriguez-Wallberg *et al*., 2021). However, some studies demonstrated that even before the introduction of hormonal therapy, transwomen sperm parameters seemed slightly altered compared to those from healthy sperm donors (Rodriguez-Wallberg *et al*., 2021; Sermondade *et al*., 2021). Different hypotheses try to explain that like physiological changes on spermatogenesis, psychological stress, androgen receptor polymorphism, genetic disorders and transgender specific factors such as intentional retraction of the testes into the groin (i.e. tucking), wearing of tight underwear to conceal the genitalia and unreported self-medication with GAHT (Rodriguez-Wallberg *et al*., 2021).

Transgender male patients can have ovarian stimulation outcomes that are similar to those of their cisgender counterparts despite having already started hormonal therapy (Leung *et al*., 2019). An interesting data was that the mean number of oocytes retrieved was higher in the transgender group than in the cisgender group (Leung *et al*., 2019). Probably because the transgender patients undergoing ovarian stimulation is younger than that of the infertility population, since the transition most of times begin during adolescence (Leung *et al*., 2019). A biological explanation for that was that their biochemical environment is similar to a woman with PCOS due to their higher levels of circulating androgens, and the androgen excess can accelerate the growth of early follicular development while slowing the rate of atresia of early antral follicles (Leung *et al*., 2019).

Although the majority of specialists agree that fertility preservation options should be provided early during the transition due to impairment of fertility promoted by the hormonal therapy, patients who have already started the transition process using hormone therapy still have the opportunity to preserve fertility (Leung *et al*., 2019; Sermondade *et al*., 2021). It is feasible and effective to provide fertility preservation for trans women through sperm cryopreservation and in trans men through oocyte or embryo cryopreservation with excellent results (Leung *et al*., 2019; Sermondade *et al*., 2021). Although further use of cryopreserved gametes remains uncertain and will depend on current regulations in various countries, the cryopreservation of gametes represents an important step in global care for transgender people (Sermondade *et al*., 2021). And although outcome data from patients who transferred an embryo is limited, preliminary findings suggest a high rate of success (Leung *et al*., 2019).

## CONCLUSION

LGBT couples represent a growing patient population in the field of fertility and reproductive medicine. With an increased cognizance of gender variance and social acceptance to transgender and nonbinary people, the access to medical treatment and hormonal interventions has become less challenging. Considering the increase in gender-affirming therapy, counseling LGBT patients, specially transgender patients, about fertility preservation has become an area of increasing concern. The use of gender-affirming hormone therapy for trans individuals has a detrimental impact on the potential for future fertility. However, numerous studies have reported a limited standard of care with many transgender patients who are experiencing inadequate fertility counseling. However, the aim of this review was to assist in the restructuring of healthcare services, routines and procedures, mainly related to reproductive medicine, in order to promote changes in values based on respect for differences. In conclusion, the healthcare personnel of fertility centers should undergo specific training and preparations to meet the LGBT patient population specific demands and to overcome communication barriers.
